# Mosquitoes LTR Retrotransposons: A Deeper View into the Genomic Sequence of *Culex quinquefasciatus*

**DOI:** 10.1371/journal.pone.0030770

**Published:** 2012-02-24

**Authors:** Renè Massimiliano Marsano, Daniela Leronni, Pietro D'Addabbo, Luigi Viggiano, Eustachio Tarasco, Ruggiero Caizzi

**Affiliations:** 1 Dipartimento di Biologia - Sezione di Genetica e Microbiologia, Università di Bari “Aldo Moro”, Bari, Italy; 2 Sezione di Entomologia e Zoologia, Dipartimento di Biologia e Chimica Agro-Forestale ed Ambientale, Università di Bari “Aldo Moro”, Bari, Italy; University of Oxford, United Kingdom

## Abstract

A set of 67 novel LTR-retrotransposon has been identified by *in silico* analyses of the *Culex quinquefasciatus* genome using the LTR_STRUC program. The phylogenetic analysis shows that 29 novel and putatively functional LTR-retrotransposons detected belong to the Ty3/gypsy group. Our results demonstrate that, by considering only families containing potentially autonomous LTR-retrotransposons, they account for about 1% of the genome of *C. quinquefasciatus*. In previous studies it has been estimated that 29% of the genome of *C. quinquefasciatus* is occupied by mobile genetic elements.

The potential role of retrotransposon insertions strictly associated with host genes is described and discussed along with the possible origin of a retrotransposon with peculiar Primer Binding Site region. Finally, we report the presence of a group of 38 retrotransposons, carrying tandem repeated sequences but lacking coding potential, and apparently lacking “master copy” elements from which they could have originated. The features of the repetitive sequences found in these non-autonomous LTR retrotransposons are described, and their possible role discussed.

These results integrate the existing data on the genomics of an important virus-borne disease vector.

## Introduction

Transposable elements are ubiquitous component of eukaryotic genomes and, besides their mutagenic role [1], they are considered as the major source of variability that can change genomes and their expression, either considering short term or large evolutionary scale time. The action exerted by transposable elements on genomes is predominantly described in studies performed in insect where the abundance of both active and inactive forms of mobile elements have shaped their genomes structurally, functionally and evolutionarily.

The post-genomic era offers a great opportunity to shed light on the evolution of mobile genetic elements with respect to eukaryotic genome. The results obtained from several genomic studies allow the comparison of related sequences from different organisms. In addition, the great amount of sequence data produced have led to the identification of novel families of mobile genetic elements and posed a problem concerning their classification [2], [3]. Looking at their transposition mechanism, transposons can be classified into two main classes [4]. Class I elements, or retrotransposons, reverse transcribe a RNA intermediate into cDNA molecules, which is then inserted in the genome. Class I elements can be further categorized in LTR- and non-LTR retrotransposons depending on the presence or absence of direct terminal repeats. Retrotransposons are major components of eukaryotic genomes; they are among the strongest evolutionary driving force acting on the genomes [5], and are potentially able to change gene expression patterns [6] [7]. Their ability to inflate eukaryotic genome size [8] is also at the basis for their use as molecular markers in organisms of socio-economic interest [9].

In the last years the rising interest in the field of mosquitoes' genomics is demonstrated by the completion of three genome sequences, and this mainly comes from their role as vectors of virus-borne diseases.

Three mosquitoes' genomes have been sequenced and assembled to date. The first mosquito genome to be sequenced was the *Anopheles gambiae* [10] followed by the sequencing of the *Aedes aegypti*'s genome [11].

*Culex quinquefasciatus* is the main vector of the nematode *W. bancrofti*, one of the known causes of the lymphatic filariasis, and its genome (about 540 Mbp) [12] has been recently sequenced [13]. Among the Culicidae family, the Anophelinae and the Culicinae subfamilies have diverged about 145–200 Mya, while within the Culicinae subfamily, Aedes and Culex genera have diverged about 52–54 Mya [13]. With this effort, a solid genomic platform for mosquito comparative genomics has been established.

Few Culex transposon families have been described in reports published before the publication of the Culex genome paper, being limited to few DNA transposon [14] [15] and retrotransposon [16] [17] [18] families.

The genomic sequence analysis performed by Arensburger et al. [13] has revealed that nearly 30% of the Culex genome is composed of TEs. Compared with the TEs content in the genomes of *A. gambiae* (16%) and *A. aegypti* (50%), this appears to be an intermediate value, as well as intermediate is the genome size of *C. quinquefasciatus* compared to the above mentioned genomes (286 Mbp and 1,3 Gbp respectively). The LTR retrotransposons identified and described in the genome sequencing paper have been deposited in the *TEfam* database [19], a specialized database for transposable elements retrieval and analyses, which focus on mosquito species. In its *Culex quinquefasciatus* section *TEfam* contains 81 families of *Bel/Pao* elements, 32 families of *Ty1/copia* elements and 57 families of *Ty3/gypsy* elements in addition to 179 families of non-LTR retrotransposons, 32 families of “cut and paste” transposons families, 3 helitrons families and 100 MITEs families.

A novel class of mobile elements with striking features has been previously described in *C. quinquefasciatus*. *Twin* is a family of atypical SINE elements with a dimer-like structure similar to a tRNA gene. It has been proposed that *Twin* family is probably a moderately repetitive sequence specific of the genus Culex, as it is absent in the genome of Aedes species [20].

Furthermore we have recently described a family of *Osvaldo*-like elements with peculiar structure of the LTRs [17].

Here, we report the presence of twenty-nine families of LTR retrotransposons in the genome of *C. quinquefasciatus*, identified using the LTR_STRUC program [21] and not reported in the *TEfam* database. One of these elements has an atypical Primer Binding Site region probably generated by the insertion of a tRNA dimer immediately downstream the 5′ LTR. Furthermore we have identified a group of 38 families probably composed of non-autonomous elements, apparently unrelated to any known retrotransposon family, which contain tandem repeated sequences between the LTRs.

The results of the genomic distribution analysis show that the novel retrotransposons identified in this paper are preferentially located in intergenic regions or in intron sequences in the genome of *C. quinquefasciatus*. Several insertions that may potentially contribute to the organization of protein-coding genes have been identified. The possible functional role of these insertions on the host gene organization is discussed.

## Materials and Methods

### LTR_STRUC analysis and classification of LTR retrotransposons

The genome sequence of *C. quinquefasciatus* was downloaded from the Broad Institute website (http://www.broad.mit.edu/index.html) and scanned with the LTR_STRUC program [21] using the default parameters. 1179 putative retrotransposon sequences obtained as output were subjected to an “all against all” BLAST in order to group sequences with % identity greater than 98% over a sequence of at least 1 Kb. 157 groups containing at least one sequences were obtained after this step. The final subset of LTR retrotransposons was then BLAST-searched against the TEfam database in order to define families and to highlight previously not annotated sequences. In order to confirm the results obtained by LTR_STRUC we have performed a LTR-retrotransposon search using the LTRharvest program [22]. The results obtained were compared to the TEfam database and the LTR_STRUC output.

Criteria for defining LTR-retrotransposons were identical to the previously described criteria adopted during ***A. aegypti*** TE analysis [17]. Briefly, sequences of the *Ty3/gypsy* LTR retrotransposons are considered as belonging to the same element if they share at least 85% nucleotide identity along at least 400 bp in their coding region. *Ty1/Copia* sequences that share at least 85% identity at the nucleotide level over at least 1000 bp are considered belonging to the same element. Copies of *Pao*/*Bel* retrotransposons are considered as belonging to the same element if they show at least 70% identity at the nucleotide level in their coding sequences.

The names assigned to the newly discovered retrotransposons follow the nomenclature adopted in the Repbase database [23] and contain the prefix “Cq” for species (Culex) and genera (quinquefasciatus), the specification of the family (namely Ty3/gypsy, Ty1/copia, BEL, etc.) and a number suffix.

### Analysis of insertions

The ORF finder program (http://www.ncbi.nlm.nih.gov/gorf/gorf.html) was used to determine the ORF number of each element detected.

The TSD (Target Site Duplicated upon insertion) and the length of the LTRs of each element obtained were determined by visual inspection of sequences. In absence of a reported list of the tRNA gene sequences in *C. quinquefasciatus* the PBS sequences were determined by comparison of a tRNA dataset of *A.gambiae* at the http://lowelab.ucsc.edu/GtRNAdb/Agamb/ website. The tRNA genes of *A. gambiae* are highly similar (if not identical in most of the cases) to the tRNA of *C. quinquefasciatus*, as demonstrated by BLAST analysis (not shown). This data ensure that a good PBS prediction has been done using the *A. gambiae* tRNA dataset.

To detect retrotransposon insertions near (or overlapping) host genes, a BLAST search at the Vectorbase database (http://www.vectorbase.org/) was performed using the following arbitrary criteria: 1) only insertions with average similarity greater than 85% were counted; 2) insertions shorter than 180 bp were not taken in account; 3) the E value was lower than 1E^−40^. These criteria allow the detection of full-length elements and defective elements without missing solo LTR and preventing misleading results coming from low quality alignments. The analyses of tandem repeats contained into retrotransposon were performed with the Tandem Repeat Finder program [24] using the basic option.

### RepeatMasker analysis

RepeatMasker software (version 3.2.9) [25] was used to estimate the retrotransposons occupancy as percent of the genome fraction. Repeats search was performed using Cross_Match as sequence search engine. A repeats library was built starting from the LTR retrotransposon group described in this paper (file S1), and it was used to scan the genome sequence. Scanning was carried out using a cutoff value of 250.

### Multiple sequence alignment and phylogenetic analysis

As previously described [26] the best way to reconstruct phylogeny of retroelements is to perform multiple alignment of RT-RnaseH-INT domains. These domains encoded by each putatively active element were extracted from the translated ORF encoding the POL polyprotein and used to reconstruct the phylogenetic history of ***C. quinquefasciatus*** Ty3/gypsy like retrotransposons. We have no evidence of domain swapping by performing multiple alignment using RnaseH, RT or INT domains (data not shown) at least for the elements analyzed in this paper. Either MUSCLE [27] or ClustalX [28] were used to perform multiple alignments. After a manual check of the alignments Neighbor-joining tree with bootstrap analyses were generated using MEGA5 [29]. As reference, previously described elements in other species [19] [17] [26] were used to establish relationships between ***C. quinquefasciatus*** retroelements.

Multiple alignments are available as file S2.

## Results

The genome sequence of *C. quinquefasciatus* (assembly version CpipJ1) was analyzed using the LTR_STRUC program, in order to obtain LTR-retrotransposon sequences.

The 1179 insertions obtained were clustered into groups of nearly identical sequences (see Material and Methods section). This allowed the identification of 157 families of elements containing at least one retrotransposon copy. The DNA sequence of representative elements of each family was BLAST-searched against the *TEfam* database. Only sequences that did not match any of the elements reported in TEfam were further analyzed. This led to the identification of 29 previously not described and potentially active elements (i.e. containing the genetic specification for the transposition machinery and the required *cis*-acting sequences). A single representative element of each family was used in the phylogenetic analysis. Representative elements were chosen among those having the best match between the two LTRs, the longest sequence and the simplest ORF structure, coding for the entire set of protein domain typically found in the family. Furthermore, elements with such features could be potentially functional and transpositionally active. Although we have identified *Ty1-copia* and *Bel-Pao* elements, they were not further analyzed due to the presence of identical sequences in the TEfam database.

**Figure 1:**
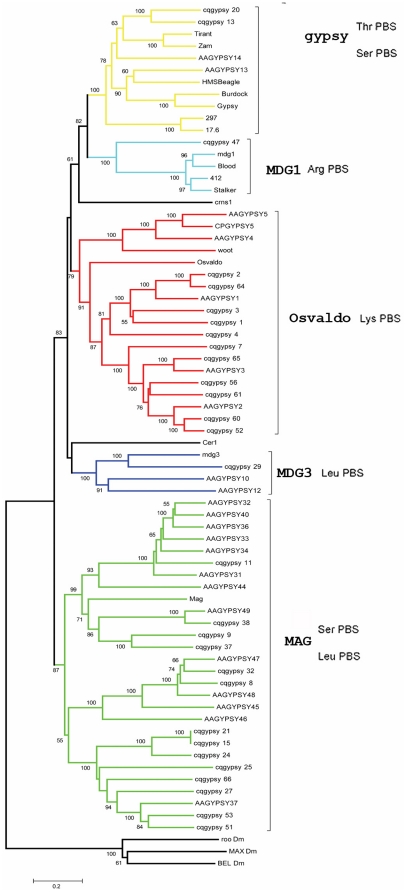
Evolutionary relationships of *C. quinquefasciatus* LTR-retrotransposons. Phylogenetic relationships of the LTR retrotransposons based on the amino acids alignment of the conserved RT, RNase H and INT domains. The clades in which fall retrotransposons detected in this paper are indicated with different colors, along with the most common tRNA complementary to the PBS is indicated for each homogeneous group. Elements from this study are indicated as “cpgypsy_” followed by a number. AAGYPSY# elements are LTR retrotransposons identified in previous analyses [17]. The N-J bootstrap values supporting the internal branches are indicated at the nodes. Only bootstrap values greater than 50% are reported. Bel-like elements were used as outgroup. Note that, for families composed of two or more copies (see table 1), representative elements (see file S1) were used for the phylogenetic analyses.

A phylogenetic analysis was performed in order to identify the origin of each group of sequences extrapolated from the LTR_STRUC output. The RT-RNaseH-INT domains of the POL polyproteins were aligned along with the corresponding domains of reference elements. This multiple alignment was then used to generate a NJ tree. As can be observed in figure 1, all the novel elements identified fall into the *Ty3-gypsy* superfamily of LTR-retrotransposons. Furthermore, the results reported in figure 1 clearly show that the new elements reported belong to five distinct lineages (namely *gypsy*, *Osvaldo*, *Mag mdg3* and *mdg1*). No novel *CsRn1*-like elements were detected despite they are well represented in the genome of *C. quinquefasciatus*, as demonstrated by the presence of nine *CsRn1*-like elements in the TEfam database.

The structural features of the retrotransposons identified in this study were also analyzed and reported in table 1. Except for few cases that will be discussed below, the main features of these elements (namely PBS type and LTR mean length) are in agreement with those of known elements belonging to the same lineage and described in other species. In table 1 is also reported the percent nucleotide identity between the LTR of each insertion detected. This value gives an approximate idea of the age of the insertions. To the best of our knowledge, the synonymous substitution rate has not been estimated for *C.quinquefasciatus*; consequently we are not able to make more precise estimations of the age of insertions.

**Table 1:** Structural features of the *C. quinquefasciatus* LTR-retrotransposons detected.

Lineage	Family	copies	Element	length	LTRs	%LNI	ORFs	PBS	TSD	supercont
Mag	cqgypsy_8	3	cqgypsy_8.1	4993	179	100	1	Leu	gtcac	3.1653
*Mag*	*cqgypsy_9*	*1*	*cqgypsy_9.1*	*4568*	*145*	*100*	*1*	*Leu*	*tttag*	*3.1194*
Mag	cqgypsy_11	11	cqgypsy_11.1	5129	169/181	99	2	Ser	ataa	3.429
*Mag*	*cqgypsy_15*	*4*	*cqgypsy_15.1*	*4851*	*197*	*99*	*1*	*Ser*	*tccag*	*3.1361*
Mag	cqgypsy_21	2	cqgypsy_21.1	6184	287	99	2	Ser	tcctt	3.770
*Mag*	*cqgypsy_24*	*6*	*cqgypsy_24.1*	*10446*	*304*	*99*	*2*	*Ser*	*accag*	*3.163*
Mag	cqgypsy_25	6	cqgypsy_25.1	7859	198	99	2	Arg	ggaag	3.176
*Mag*	*cqgypsy_27*	*4*	*cqgypsy_27.1*	*5260*	*196*	*97*	*2*	*Ser*	*gtgcc*	*3.790*
Mag	cqgypsy_32	3	cqgypsy_32.1	4918	190	99.5	1	Leu	ggaat	3.540
			cqgypsy_32.2	4779	182	97.8	3	Leu	attac	3.1290
*Mag*	*cqgypsy_37*		*cqgypsy_37.1*	*4078*	*139/143*	*92.4*	*1*	*Ser*	*cttgc*	*3.100*
			*cqgypsy_37.2*	*9065*	*152*	*98.7*	*1*	*Ser*	*ataat*	*3.30*
Mag	cqgypsy_38	5	cqgypsy_38.1	4472	164	97.6	2	Ser	cctgg	3.723
			cqgypsy_38.2	4534	164	97.6	2	Ser	ttaat	3.1068
			cqgypsy_38.3	3248	119	97.3	2	Ser	attcc	3.1314
*Mag*	*cqgypsy_53*	*31*	*cqgypsy_53.1*	*5310*	*211*	*97.2*	*1*	*Ser*	*cactt*	*3.144*
			*cqgypsy_53.2*	*2887*	*211/213*	*99.1*	*frag*	*Ser*	*aggac*	*3.1107*
Mag	cqgypsy_51	5	cqgypsy_51.1	4904	179	100	2	Ser	acctg	3.1151
			cqgypsy_51.2	6291	179	98.9	2	Ser	gacac	3.243
			cqgypsy_51.3	4575	188	100	frag	Ser	aacac	3.1291
*Mag*	*cqgypsy_66*		*cqgypsy_66.1*	*7544*	*208*	*100*	*2*	*Ser*	*ctatt*	*3.7*
Gypsy	cqgypsy_13	7	cqgypsy_13.1	7249	302	100	3	Thr	tatata	3.734
*Gypsy*	*cqgypsy_20*	*3*	*cqgypsy_20.1*	*7438*	*357*	*100*	*3*	*Ser*	*atata*	*3.1285*
Mdg3	cqgypsy_29	4	cqgypsy_29.1	5316	264	100	2	Leu	gttg	3.462
			cqgypsy_29.2	5343	263	99.6	2	Leu	atag	3.168
*Mdg1*	*cqgypsy_47*	*25*	*cqgypsy_47.1*	*6771*	*431*	*100*	*2*	*Arg*	*cttc*	*3.2173*
			*cqgypsy_47.2*	*8540*	*444*	*100*	*1*	*Arg*	*gaac*	*3.13*
			*cqgypsy_47.3*	*6623*	*444*	*100*	*2*	*Arg*	*ccac*	*3.33*
			*cqgypsy_47.4*	*6751*	*432*	*99.3*	*2*	*Arg*	*cagg*	*3.508*
			*cqgypsy_47.5*	*6772*	*431*	*99.9*	*2*	*Arg*	*gccg*	*3.346*
Osvaldo	cqgypsy_1	3	cqgypsy_1.1	11926	2137	99	2	Lys	ggtt	3.62
*Osvaldo*	*cqgypsy_2*	*21*	*cqgypsy_2.1*	*12138*	*2055/2056*	*99*	*2*	*Lys*	*aact*	*3.1399*
			*cqgypsy_2.2*	*5491*	*2054/2057*	*99.7*	*frag*	*Lys*	*tgct*	*3.72*
Osvaldo	cqgypsy_3	7	cqgypsy_3.1	10049	1591/1596	99	2	Lys	aagt	3.349
*Osvaldo*	*cqgypsy_4*	*6*	*cqgypsy_4.1*	*10581*	*1742*	*99*	*2*	*Lys*	*caac*	*3.169*
Osvaldo	cqgypsy_7	5	cqgypsy_7.1	6914	997	99	1	Lys	aagt	3.38
*Osvaldo*	*cqgypsy_52*	*29*	*cqgypsy_52.1*	*10354*	*1340*	*99.6*	*1*	*Lys*	*caaa*	*3.458*
			*cqgypsy_52.2*	*7373*	*1345/1346*	*99.4*	*frag*	*Lys*	*agct*	*3.70*
Osvaldo	cqgypsy_56	14	cqgypsy_56.1	9473	1384	100.	2	Lys	aaat	3.568
			cqgypsy_56.2	9393	1369/1368	95.1	2	Lys	ttat	3.83
*Osvaldo*	*cqgypsy_61*	*15*	*cqgypsy_61.1*	*9196*	*1151*	*99.6*	*2*	*Lys*	*caaaag*	*3.1285*
			*cqgypsy_61.2*	*10261*	*246*	*97*	*frag*	*Lys*	*attat*	*3.330*
Osvaldo	cqgypsy_60	48	cqgypsy_60.1	10172	1308	99.3	2	Lys	acaac	3.133
			cqgypsy_60.2	10164	1310	99.9	frag	Lys	actt	3.784
			cqgypsy_60.3	9985	1224	99.6	2	Lys	cagg	3.215
*Osvaldo*	*cqgypsy_64*	*41*	*cqgypsy_64.1*	*12479*	*2045/2046*	*99.8*	*2*	*Lys*	*acgt*	*3.82*
			*cqgypsy_64.2*	*12368*	*2038/2037*	*98.6*	*2*	*Lys*	*aagc*	*3.191*
			*cqgypsy_64.3*	*8014*	*2047/234*	*98.*	*frag*	*Lys*	*agat*	*3.141*
			*cqgypsy_64.4*	*7680*	*1857*	*100*	*frag*	*Lys*	*ctat*	*3.82*
Osvaldo	cqgypsy_65	20	cqgypsy_65.1	10447	1565	99%	2	Lys	aacc	3.254

“Lineage” indicates the major lineage they belong to; the estimated copy number detected by BLAST analysis is indicated in the column “copies”; copies enumerated in column “Elements” are those identified by the LTR_STRUC program; “length” indicates the overall element length; “ORFs” indicates the number of ORFs detected in each element; TSD shows the target sequence duplicated upon insertion, Primer Binding Site (PBS); LTR indicates the LTR length; supercontig indicates the supercontig where a given element was identified. %LNI: percent LTRs nucleotide identity.

Note that two values are reported in the LTRs column if the two LTRs of an element differ in size. “frag” indicates fragmented coding regions.

No target site preference was observed for any of the retrotransposons analyzed.

A closer view of the phylogenetic analysis results indicates that eleven elements can be classified as *Osvaldo*-like, two fall in the *gypsy* lineage, one in the *Mdg1* and *Mdg3* lineages respectively. The analysis performed was aimed to dissect the structural properties for each family detected, and to compare them with those of known elements of the same phylogenetic lineage.

### *Gypsy* lineage

Two novel *gypsy*-like elements have been identified in this study. The structural analyses have revealed that the first base of the putative PBS overlaps the last base of the 5′ LTR in these elements; this was also observed for the *gypsy* element of *D. melanogaster* [30]. It can be assumed that this a general rule for the members of the *gypsy* lineage identified in other organisms.

Several members of the gypsy lineage identified so far in other organisms contain an ORF that could potentially encode for the envelope protein (ENV), a typical retrovirus like protein reported to be important in the horizontal transmission process [31]. The two *gypsy*-like elements detected in this study also contain an ORF that potentially encodes an ENV-like protein. The conceptual translation of these putative env-coding regions reveals typical domains of ENV proteins (not shown).

### *Mag* lineage

Members of this lineage have been previously identified in several insect genomes such as *B. mori* [32], *A. gambiae*, *D. melanogaster* [33] and *C. elegans* [34] [35].

Thirteen families are phylogenetically related to the *Mag* element. The PBS of the *Mag*-like elements identified is complementary either to the tRNA^Leu^ or to the tRNA^Ser^. A single element (*cqgypsy_25*) with an atypical PBS sequence, complementary to the tRNA^Arg^, has been identified. Three elements (namely *cqgypsy_24*, *cqgypsy_25* and *cqgypsy_66*) contain tandem repeated sequences in the 5′ UTR. The unusual size of the *cqgypsy_24* element (greater than 10 Kbp) is due to the size of a repeated region (about 3 Kbp).

The phylogenetic analysis shows that the *Mag* clade is formed by two subgroups strongly supported by high bootstrap values. Four elements of *C. quinquefasciatus* co-cluster with the *Mag* element, while 9 elements fall into the second cluster with five elements from *A. aegypti* used as reference elements.

### *Mdg1* and *Mdg3* lineages

Two elements identified in this paper belong to the *Mdg1* and *Mdg3* clades respectively. *cqgypsy_47* belongs to the *Mdg1* clade while *cqgypsy_29* belongs to the *Mdg3* lineage. Looking at the TEfam database, eight *Mdg1*-like elements and three *Mdg3*-like elements can be retrieved. This suggests the possibility that these two clades could be poorly represented in the genome of *C. quinquefasciatus*.

### *Osvaldo* lineage

Existing data in the TEfam database, suggest that these elements are abundant in the family *Culicidae*. Twenty-nine *Osvaldo*-like elements are annotated in the genome of *A. aegypti* and five elements in the genome of *C. quinquefasciatus*.

**Figure 2:**
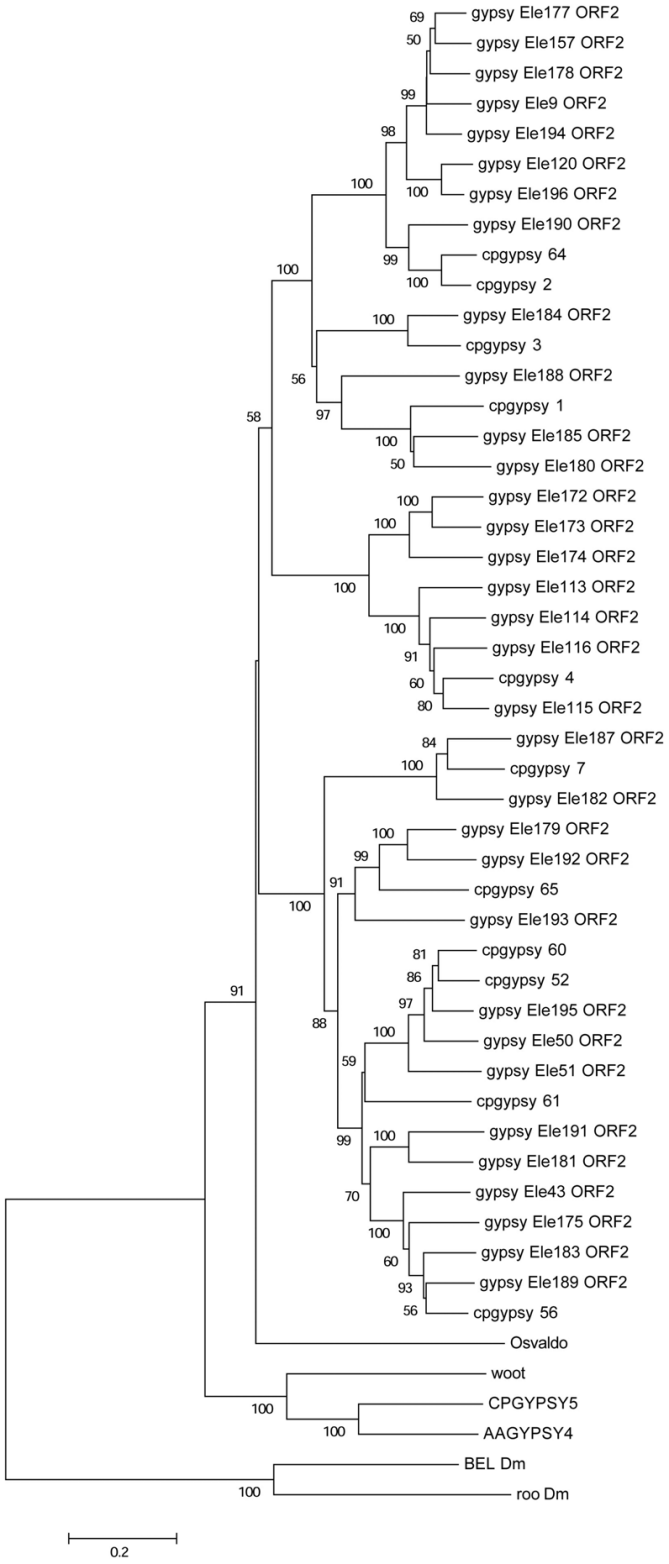
Evolutionary relationships of *Osvaldo*-like elements of *C. quinquefasciatus* LTR-retrotransposons. Phylogenetic relationships of the *Osvaldo*-like retrotransposons based on the amino acids alignment of the conserved RT, RNase H and INT domains CPGYPSY5 and AAGYPSY# are LTR retrotransposons identified in previous analyses [17]. Elements “gypsy ELE ###” were retrieved from the TEfam database. The N-J bootstrap values supporting the internal branches are indicated at the nodes. Only bootstrap values greater than 50% are reported. Bel-like elements were used as outgroup.

Querying the TEfam dataset for *Osvaldo*-like elements in *C. quinquefasciatus* results in five annotated elements. We have identified 11 unreported *Osvaldo*-like elements in the genome of *C. quinquefasciatus*. Their LTRs length ranges from 997 to 2055 bp, a feature that characterizes members of the Osvaldo lineage. In figure 2 are showed the phylogenetic relationships of the *Osvaldo*-like elements identified in *A. aegypti* and *C. quinquefasciatus*. No species-specific cluster was observed in the distribution of these elements. Copy number varies among different families of *Osvaldo*-like elements (see table 1) and the PBS is invariantly complementary to the 3′ end of the tRNA^Lys^. This is also the initiator tRNA used by *Osvaldo* [36]. As reported in our previous analyses, both genomes of *A. aegypti* and *C. quinquefasciatus* contain retrotransposons that are strictly related to the *woot* element of *T. castaneum* [17], but containing unusually short LTRs. The CPGYPSY5 element identified by Minervini et al by BLAST similarity search was also identified during the course of this analysis by the LTR_STRUC program.

**Figure 3:**
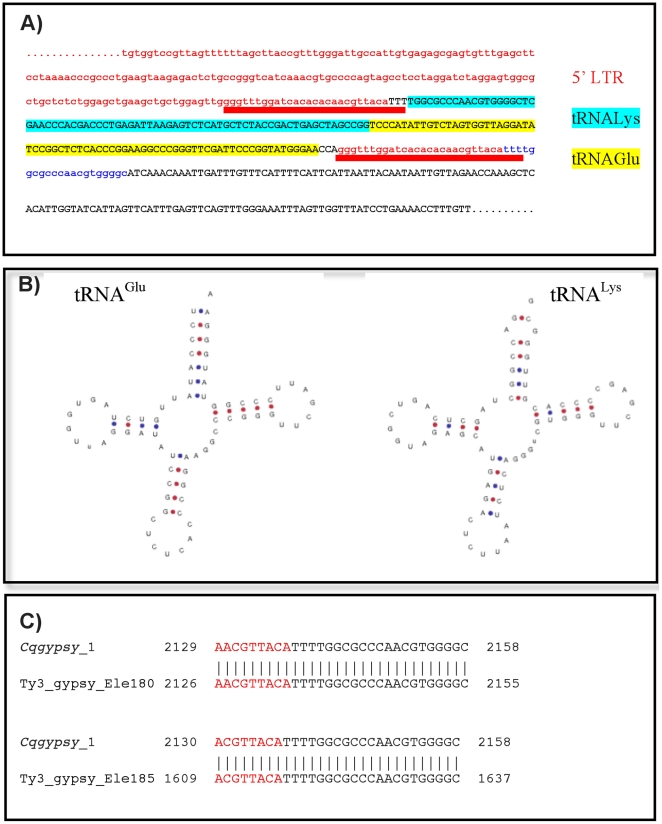
Organization of the LTR-PBS region of *cqgypsy_1*. A) The tRNA sequences inserted into the 5′LTR of the *cqgypsy_1* element. The LTR sequence is colored in red, while the PBS sequence is colored in blue. The red bar indicates the duplicated sequence surrounding the putative Twin element. Each of the tRNA halves of the putative Twin is highlighted in turquoise (tRNA^Lys^) or in yellow (tRNA^Glu^). The PBS is depicted in blue. B) tRNAscan output showing the secondary structure of the two halves of the insertion as a cloverleaf structure. C) Local alignment results of *cqgypsy_1* with the *gypsy_Ele180* and *gypsy_Ele185*. The aligned region correspond to the 5′LTR (red)/PBS(black) boundary.

*cqgypsy_1* is a peculiar element of the *Osvaldo* lineage. It has been detected as single copy retrotransposon by LTR_STRUC analysis, but probably present in multiple copies in the genome of *C. quinquefasciatus* as revealed by BLAST analyses on the trace archive (not shown). The structural analysis of its PBS region shows that it has a non-canonical PBS. Instead of a short nucleotide stretch complementary to the 3′ end of a tRNA, we have found a 149 bp long sequence identical to two tRNA arranged in a head to head fashion. The 149 bp sequence is recognized by the tRNAscan program, which in turn gives two perfectly folded tRNA molecules as output (figure 3B)

The unusual configuration of the PBS region of *cqgypsy_1* has been analyzed in details. As can be observed in figure 3, both tRNA-like sequences have the terminal CCA sequences. Furthermore a direct duplication of 26 nucleotides of the 5′LTR has been found at both sides of the tandem tRNA copies. The tandem copies of tRNA identified in *cqgypsy_1* are somehow reminiscent of the structure of *Twin* elements described by Feschotte and co-authors [20]. *Twin* has been described as a novel type of SINE element consisting of two tRNA related regions separated by a 39 bp spacer.

We have also analyzed in detail the phylogenetic relationships of *cqgypsy_1* with other elements of the *Osvaldo* lineage belonging to different mosquito genomes. Its closest relative is the *Ty3_gypsy_Ele185* and *Ty3_gypsy_Ele180* elements annotated in the TEfam database (TEfam ID TF000935 and TF000939 respectively). None of the related elements of *A. aegypti* contain such tandem copy of tRNA. We do not expect to observe significant sequence similarity at the nucleotide level when Culex and Aedes elements were compared in a pair-wise alignment, despite the strict relationship observed at the protein level. By comparing the three *Osvaldo*-like elements, *cqgypsy_1*, *Ty3_gypsy_Ele185* and *Ty3_gypsy_Ele180*, we have detected a similarity region in a 29–30 nucleotides region encompassing the boundary between the 5′LTR and the PBS region (see figure 3C), suggesting an unusually strong cross-species conservation of the LTR sequence flanking the PBS. This conservation across the 5′LTR boundary and the PBS was not observed after comparison of any of the retrotransposons analyzed in this paper with their relatives in *Aedes aegypti*. Taken together, these results confirm the phylogenetic relationship among these elements and indicate a strong conservation of the 30-nucleotide long sequence across the 5′ LTR shared by Culex and Aedes elements, which is probably under functional constrains.

### Non-autonomous elements

Non-autonomous elements are important to understand the evolutionary dynamics of transposable elements in the genomic context [37]. Non-autonomous elements were also detected and analyzed in this work. The LTR_STRUC program is also able to find aberrant retrotransposon sequences (i.e. LTR-retrotransposon with internal deletions of various size); in this case a lower score is assigned respect to a potentially active retrotransposon. However, most of the defective LTR retrotransposons detected are false positives resulting from a couple of direct repeats (mimicking the LTRs) but lacking PBS, PPT, the target site duplication and the coding sequences. A certain number of low scoring sequences extracted by LTR_STRUC are *bona fide* defective elements. Several nested elements were also found in the output of LTR_STRUC, but no significant bias of nesting was observed. In general, truncated retrotransposons are related to at least one putatively active element, in the TEfam dataset or in our output, thus falling into a specific family of elements that, for this reason, will be composed by autonomous and non-autonomous elements.

Notably, we have found a group of non-autonomous elements lacking coding sequences, and that cannot be related to any of the known putatively active elements annotated in *TEfam*, nor to any of the elements identified in this work. The features of these elements are summarized in table 2.

**Table 2:** Features of the non-autonomous LTR retrotransposons identified in this paper.

Element	supercont	length	LTR	PBS	TSD	Rep Position	Period	Copies	Entropy	%
*cqUNK_1*	*3.2*	*2994*	*261*	*Arg*	*caagg*	*583–1963*	*155*	*8.9*	*1.96*	
						*2188–2226*	*16*	*2.4*	*1.40*	*52.9*
						*2206–2380*	*22*	*8.0*	*1.40*	
cqUNK_3	3.322	14602	315/337	Leu	attcc	3252–3314	2	31.5	1.10	0.9
						13294–13367	33	2.2	1.63	
*cqUNK_4*	*3.720*	*2990*	*389*	*Arg*	*ttct*	*540–625*	*34*	*2.5*	*1.94*	*2.8*
cqUNK_5	3.49	6237	931	Asn	nd	2701–2763	12	5.3	1.28	
						2933–3723	164	4.8	1.97	14.2
						5366–5398	17	1.9	1.94	
*cqUNK_6*	*3.403*	*1629*	*125*	*Pro*	*nd*	*323–375*	*16*	*3.3*	*1.50*	
						*536–1337*	*164*	*4.9*	*1.97*	
						*2086–2447*	*129*	*2.8*	*1.75*	*94.9*
						*4245–4281*	*18*	*2.1*	*1.50*	
						*7914–7960*	*16*	*3.0*	*1.86*	
cqUNK_7	3.506	2267	182	Arg	ggtgc	608–1551	117	8.1	1.96	41.6
*cqUNK_9*	*3.65*	*4795*	*193*	*Ser*	*gatc*	*1365–1740*	*49*	*7.7*	*1.92*	*7.8*
cqUNK_10	3.176	3540	194	Ser	agaag	1278–1780	144	3.5	1.91	14.2
*cqUNK_11*	*3.710*	*2554*	*170*	*Arg*	*catt*	*1040–2015*	*298*	*3.3*	*2.00*	*38.2*
cqUNK_12	3.654	5450	280	Leu	acaag	3411–4140	88	8.3	1.94	20.1
						4176–4541	50	7.3	1.95	
*cqUNK_13*	*3.450*	*4810*	*573*	*Tyr*	*nd*	*1481–1731*	*82*	*3.0*	*1.93*	*5.2*
cqUNK_14	3.563	4105	176	Arg	ggcta	1564–3072	93	16.7	1.97	36.5
*cqUNK_15*	*3.622*	*5405*	*182*	*Arg*	*nd*	*1805–2384*	*92*	*6.3*	*1.98*	*10.7*
cqUNK_16	3.54	6178	334	Ser	nd	2066–2417	159	2.2	2.00	5.7
*cqUNK_17*	*3.456*	*5460*	*194*	*Met*	*actac*	*2105–3007*	*51*	*19.7*	*1.98*	*13.0*
cqUNK_18	3.688	4616	205	Asp	acaga	2097–2230	72	1.9	1.91	
						3114–3334	51	4.3	1.96	14.2
						3345–3646	48	6.3	1.92	
*cqUNK_19*	*3.258*	*3639*	*360/337*	*Tyr*	*aatac*	*970–1162*	*103*	*1.9*	*1.95*	*14.5*
						*1226–1561*	*154*	*2.2*	*1.95*	
cqUNK_20	3.707	6208	519	Ser	atctg	2130–2843	39	18.2	1.97	11.5
*cqUNK_21*	*3.144*	*5520*	*269*	*Ser*	*acgac*	*642–734*	*44*	*2.1*	*1.80*	
						*1286–1714*	*114*	*3.7*	*1.96*	*20.2*
						*1960–2554*	*88*	*6.8*	*1.92*	
cqUNK_22	3.589	8814	182	Ser	tactc	1373–3671	164	14.0	1.97	40.1
						5792–6779	164	6.0	1.97	
						7247–7500	27	9.7	1.91	
						7526–7852	160	2.0	1.80	
*cqUNK_23*	*3.1311*	*1909*	*193*	*Arg*	*gtaac*	*1003–1633*	*76*	*8.3*	*1.98*	*33.0*
cqUNK_24	3.393	4023	185	Ser	ttcat	401–444	9	5.1	1.40	9.2
						3370–3696	41	8.0	1.84	
*cqUNK_25*	*3.2077*	*2965*	*276*	*Met*	*ttggg*	*1389–1758*	*68*	*5.4*	*1.98*	*12.4*
cqUNK_26	3.220	3444	337	Ser	cagcc	593–2143	150	10.3	1.89	51.4
						2332–2552	73	3.0	1.64	
*cqUNK_27*	*3.124*	*4080*	*219*	*Arg*	*gcctt*	*1118–2136*	*217*	*4.7*	*1.98*	*31.1*
						*2160–2411*	*64*	*3.9*	*1.97*	
cqUNK_28	3.537	5520	197	Arg	caccc	942–1803	72	12.0	1.99	15.6
						3221–3349	65	2.0	1.69	
*cqUNK_29*	*3.172*	*3300*	*176*	*Arg*	*caagc*	*968–1807*	*78*	*10.7*	*1.97*	*25.4*
cqUNK_31	3.1198	3124	371	Ser	gtcca	1001–1586	159	3.7	1.93	31.4
						1633–2047	40	11.3	1.76	
*cqUNK_32*	*3.496*	*5091*	*318*	*Ser*	*nd*	*835–1520*	*62*	*11.0*	*1.92*	*13.4*
cqUNK_33	3.1048	5047	694	Tyr	nd	915–1217	167	1.8	2.00	6.0
*cqUNK_35*	*3.492*	*6910*	*224*	*Gln*	*nd*	*2243–5096*	*44*	*67.3*	*1.95*	*39.6*
cqUNK_37	3.343	5389	189	Met	actgg	2792–3519	179	4.1	1.99	13.5
*cqUNK_38*	*3.1148*	*7609*	*246*	*Arg*	*ggtat*	*558–812*	*74*	*3.4*	*1.94*	
						*914–1124*	*31*	*6.8*	*1.88*	*7.5*
						*1200–1303*	*31*	*3.4*	*1.88*	
cqUNK_39	3.820	5080	573/581	Tyr	tgatg	2829–2899	35	2.0	1.95	1.4
*cqUNK_41*	*3.723*	*10955*	*223*	*Ala*	*gtggt*	*3037–4288*	*408*	*3.1*	*1.92*	
						*4795–5140*	*45*	*7.6*	*1.91*	
						*9068–9146*	*39*	*1.9*	*1.64*	*17.0*
						*9288–9395*	*48*	*2.2*	*1.65*	
						*9446–9533*	*42*	*2.1*	*1.49*	
cqUNK_42	3.7	7544	208	Ser	ctatt	691–1175	127	3.9	1.90	6.4
*cqUNK_43*	*3.2654*	*6161*	*224*	*Thr*	*caagg*	*1199–3865*	*46*	*58.1*	*1.93*	*43.3*
cqUNK_45	3.590	3246	315	Gln	nd	2382–2901	278	1.9	1.99	16.0

For each non-autonomous element is reported the supercontig in which a representative element can be found, the overall length, the LTR size, the tRNA complementary to the PBS. It is also indicated the position, the period and the copies of the repeated DNA contained in the elements listed. The entropy value gives an estimation of the complexity of the repeats (see main text). The portion occupied by repeats in terms of % of the total size of the element is also indicated (column %).

Elements belonging to this group are featured by highly similar LTR sequences (>98% identity), a sharply definable PBS sequence immediately downstream the 5′LTR, a PPT upstream the 3′LTR and a duplicated sequence at the insertion site. We were unable to classify these elements using phylogenetic criteria, due to the lack of coding sequences that would enable common RT-based phylogenetic analyses.

In addition, a common feature of all these elements is the presence of tandemly repeated sequences bracketed by the retrotransposon LTRs.

The presence of repeated sequences into a retrotransposon seems to be a nearly exclusive feature of this group of elements. The exception is represented by three putatively active elements belonging to the *Mag* lineage (*cqgypsy_24*, *cqgypsy_25* and *cqgypsy_66*), carrying tandemly repeated sequences, identified during the genome wide screening in *C. quinquefasciatus*. Moreover, the exceptional size of these three *Mag*-like elements is due to the presence of repeats.

The repeated region sequence varies among families, and constitute as much as 95% of the entire length of a given element. Tandem repeats Finder [24] allows the estimation of the entropy value for a given DNA sequence, a parameter based on the percent base composition and whose value is comprised between zero (indicating low sequence complexity) and two (indicating high sequence complexity). A base composition analysis of the repeated sequences in these LTR-retrotransposons suggests that only in few cases they are composed by simple di-nucleotide iterations (i.e. cqUNK_3, first repeat), while in most of the cases repeats are complex stretches of DNA as demonstrated by entropy values very close to two (table 2).

LTR-retrotransposons containing repeats have been so far identified in other species [38]. Such repeats are usually located in the 5′ UTR or in the 3′ UTR of these retroelements. It has been demonstrated that tandem repeated sequences carried by retrotransposons of *Drosophila melanogaster* could behave as powerful regulatory sequences, such as enhancers of the gene expression or genetic insulators. As an example, the tandem repeat in the 5′UTR of *gypsy* is a powerful insulator [39].

Retrotransposon lacking coding sequences and not relatable to any known master copy have been also identified in *A. gambiae* in previous genome wide searches (Marsano RM unpublished results). Unlike the non-autonomous elements identified in *C. quinquefasciatus* and described above, those identified in *A. gambiae* do not contain tandemly repeated sequences.

### Distribution of the retrotransposons in the genome of *C. quinquefasciatus*

We have performed distribution analysis at the genomic level using BLAST and RepeatMasker [25]. RepeatMasker allows a rapid estimation of the genomic fraction occupied by the sequences analyzed. The analysis was performed using a custom library of repeats identified in this paper.

The genome fraction occupied by the retrotransposon sequences showed in table 1 is 0.82% (4,75 Mbp/579 Mbp). This is likely to be an underestimation due to the criteria used (see materials and methods section). Furthermore, we have intentionally excluded from this analysis the defective retrotransposons described in the previous paragraph, as they could inflate the genomic fraction due to the presence of tandem repeats, which can be found as part of complex satellite rather than retrotransposons.

The BLAST search was performed against *C. quinquefasciatus* genomic database in order to discriminate among insertions in gene free (or intergenic) genomic regions. A great number of insertions are represented by rearranged elements and by solo-LTRs that can be generated by homologous recombination events between the 5′ and 3′ LTRs.

It has been reported that several families of *gypsy*-like elements are loaded with potent regulatory elements such as enhancers [40], and insulators [41]. Such cis-regulatory elements, when brought in proximity of genes by mean of novel insertions, are able to modify their original expression pattern, in a way that is dependent of the strength of the regulatory element carried by the retrotransposon and of the distance from the endogenous gene. In order to define the distance occurring between LTR-retrotransposons and nearby genes, we performed our analysis using an arbitrary window length of 5 Kb upstream the transcriptional start site or 5 Kbp downstream the termination of transcription of genes annotated in Vectorbase and in which insertions have been detected. This analysis also enables to know if there is a contribution in the gene organization and evolution in *C. quinquefasciatus*.

Due to the large number of BLAST hits (more than 7000) obtained by searching non-autonomous elements against the genomic sequence, we have performed the BLAST search against the transcripts database and considering only insertions in the coding region of predicted genes. The results of these analyses are reported in table 3 and table 4.

**Table 3:** The contribution of LTR-retrotransposons to *C. quinquefasciatus* gene organization.

Element	Interaction	Description	GENE ID	Supercont:position
Cqgypsy_2	Within intron	Dual specificity tyrosine-phosphorylation-regulated kinase	CPIJ004687	3.72: 556,787–583,090
	Exon-Intron junction	5′-3′ exoribonuclease, putative	CPIJ016423	3.746: 154,787–166,602
	1–2 Kbp upstream	fimbrin/plastin	CPIJ004008	3.57: 387,682–393,342
	2–3 Kbp downstream	allergen, putative	CPIJ018993	3.1504: 58,993–71,374
	0–1 Kbp downstream	Adenylyltransferase and sulfurtransferase MOCS3	CPIJ001621	3.19: 246,047–247,568
*Cqgypsy_3*	*0–1 Kbp downstream*	*disulfide oxidoreductase*	*CPIJ018966*	*3.1505: 2,834–13,165*
Cqgypsy_5	1–2 Kbp downstream	chaperonin	CPIJ013429	3.475: 239,574–242,207
	Exon-Intron junction	40 S ribosomal protein S2	CPIJ012693	3.480: 10,936–19,727
	4–5 Kbp upstream	serine threonine-protein kinase	CPIJ018896	3.1443: 17,857–21,694
*Cqgypsy_7*	*Within intron*	*Brahma associated protein 170 kD, putative*	*CPIJ002241*	*3.30: 656,859–677,280*
	*Overlap first exon*	*ribosomal protein L23a*	*CPIJ016489*	*3.858: 52,166–53,573*
Cqgypsy_8	1–2 Kbp upstream	suppressor of ty3	CPIJ014381	3.539: 307,272–309,528
	1–2 Kbp downstream	suppressor of ty3	CPIJ014381	3.539: 307,272–309,528
	intron	suppressor of ty3	CPIJ014381	3.539: 307,272–309,528
*Cqgypsy_13*	*0–1 Kbp downstream*	*transcription factor IIIB 90 kDa subunit*	*CPIJ008270*	*3.167: 169,563–182,965*
Cqgypsy_15	Within intron	dystrophin major muscle isoform	CPIJ013032	3.423: 50,593–185,416
*Cqgypsy_20*	*0–1 Kbp downstream*	*histone-lysine n-methyltransferase*	*CPIJ000732*	*3.6: 1,790,053–1,797,972*
Cqgypsy_21	0–1 Kbp upstream	flotillin-2	CPIJ007626	3.148: 169,798–180,783
*Cqgypsy_25*	*Exon-Intron junction*	*phd finger protein*	*CPIJ014131*	*3.545: 75,916–92,572*
Cqgypsy_29	3–4 Kbp downstream	protein phosphatase-1	CPIJ008212	3.168: 687,712–708,602
	0–1 Kbp downstream	helicase	CPIJ019431	3.1585: 41,788–51,610
*Cqgypsy_37*	*1–2 Kbp downstream*	*sphingomyelin synthetase*	*CPIJ002233*	*3.30: 541,656–542,535*
	*2–3 Kbp downstream*	*DEAD box ATP-dependent RNA helicase*	*CPIJ006204*	*3.118: 567,640–586,793*
Cqgypsy_47	2–3 Kbp downstream	sodium/iodide cotransporter	CPIJ002364	3.33: 789,851–792,666
	4–5 Kbp upstream	serine protease inhibitor, serpin	CPIJ012013	3.346: 385,437–397,533
	3–4 Kbp downstream	pre-mrna splicing factor prp17	CPIJ011807	3.365: 424,340–426,137
	1–2 KbpUpstream	uridine cytidine kinase i	CPIJ016204	3.736: 44,070–45,560
	1–2 Kbp downstream	uridine cytidine kinase i	CPIJ016204	3.736: 44,070–45,560
	1–2 Kbp downstream	coatomer	CPIJ014834	3.606: 201,807–202,272
	1–2 Kbp downstream	poly a polymerase	CPIJ014835	3.606: 205,341–209,830
*Cqgypsy_51*	*1–2 Kbp upstream*	*zinc finger protein*	*CPIJ009854*	*3.243: 281,133–285,737*
	*2–3 Kbp upstream*	*DNA replication licensing factor MCM7*	*CPIJ009855*	*3.243: 295,787–306,221*
	*2–3 Kbp downstrean*	*mitochondrial 39 S ribosomal protein L3*	*CPIJ017407*	*3.941: 86,591–87,964*
	*1–2 Kbp downstream*	*26 S protease regulatory subunit 6a*	*CPIJ017405*	*3.941: 78,697–79,175*
Cqgypsy_53	3–4 Kbp downstream	esterase B1 precursor	CPIJ016336	3.777: 170,027–172,021
	4–5 Kbp upstream	ATP synthase D chain, mitochondrial	CPIJ011691	3.328: 179,978–180,887
	2–3 Kbp downstream	Eftud2 protein, putative	CPIJ000064	3.1: 1,221,757–1,228,225
	1–2 Kbp upstream	cell division protein kinase 5	CPIJ000065	3.1: 1,233,232–1,234,222
	Exon-Intron junction	sarcolemmal associated protein-2, putative	CPIJ011313	3.310: 56,964–69,006
*Cqgypsy_56*	*Exon-Intron junction*	*semaphorin*	*CPIJ001593*	*3.17: 1,304,933–1,339,494*
	*Exon-Intron junction*	*microfibrillar-associated protein, putative*	*CPIJ020039*	*3.2342: 8,606–17,792*
	*Exon-Intron junction*	*male-specific doublesex protein*	*CPIJ004057*	*3.59: 681,384–685,772*
	*1–2 Kbp downstream*	*polypeptide of 976aa, putative*	*CPIJ018525*	*3.1222: 14,705–19,317*
Cqgypsy_59	1 Kbp upstream	negative elongation factor E	CPIJ000025	3.1: 727,264–728,238
	4–5 Kbp downstream	semaphorin	CPIJ000027	3.1: 744,915–761,147
	4–5 Kbp downstream	superoxide dismutase, putative	CPIJ005173	3.91: 822,776–823,755
	Within intron	enhancer of polycomb	CPIJ018246	3.1131: 70,175–80,884
*Cqgypsy_60*	*Overlaps last exon*	*serine protease inhibitors putative*	*CPIJ007021*	*3.133: 747,858–748,701*
	*0–1 Kbp upstream*	*serine protease inhibitor*	*CPIJ007023*	*3.133: 758,924–759,336*
	*1 Kbp downstream*	*nucleoporin*	*CPIJ013031*	*3.426: 362,618–363,727*
	*0–1 Kbp upstream*	*40 S ribosomal protein S14-A*	*CPIJ012110*	*3.400: 69,850–70,191*
Cqgypsy_61	Overlaps last exon	cytochrome c oxidase subunit I	CPIJ016836	3.816: 17,796–33,791
*Cqgypsy_63*	*Within intron*	*Dual specificity tyrosine-phosphorylation-regulated kinase*	*CPIJ004687*	*3.72: 556,787–583,090*
	*Exon-Intron junction*	*5′-3′ exoribonuclease, putative*	*CPIJ016423*	*3.746: 154,787–166,602*
	*4–5 Kbp upstream*	*alanine-glyoxylate aminotransferase*	*CPIJ006409*	*3.128: 202,128–218,875*
Cqgypsy_64	Exon-Intron junction	arsenite inducible RNA associated protein aip-1	CPIJ005006	3.82: 520,914–524,059
	Exon-Intron junction	nk homeobox protein	CPIJ019260	3.1511: 40,639–44,852
	2–3 Kbp downstream	60 S ribosomal protein L7	CPIJ017548	3.961: 25,320–28,131

For each insertions detected in proximity (+/− 5 Kbp) or into genes are reported the kind of interaction (upstream, downstream, exon, intron), the Vectorbase identifier of the gene, its description and its position in the supercontig.

**Table 4:** Contribution of the non-autonomous elements identified in this paper to the formation of mature mRNAs of *C. quinquefasciatus* genes.

Element	Description	gene ID	Supercontig:position
CqUNK_3	ATP-dependent RNA helicase DHX8	CPIJ011263	3.322: 64334–68170
	cell cycle control protein cwf8	CPIJ011261	3.322: 54937–56710
	tRNA methyltransferase	CPIJ011262	3.322: 62963–64258
	carboxylesterase-6	CPIJ006908	3.137: 13520–18988
	bombesin receptor subtype-3	CPIJ017637	3.980: 85049–99690
	N-acetylgalactosaminyltransferase 7	CPIJ014647	3.660: 5557–7406
	saposin	CPIJ014133	3.545: 130173–138797
*CqUNK_5*	*bombesin receptor subtype-3*	*CPIJ017637*	*3.980: 85049–99690*
CqUNK_9	dopamine beta hydroxylase	CPIJ019622	3.1797: 2222–17524
*CqUNK_11*	*midasin*	*CPIJ010145*	*3.251: 518823–535901*
	*sterol desaturase, putative*	*CPIJ009637*	*3.227: 524825–527033*
CqUNK_19	malate dehydrogenase	CPIJ015299	3.611: 78722–91118
	igf2 mRNA binding protein, putative	CPIJ011349	3.312: 412154–436319
*CqUNK_20*	*regulator of chromosome condensation*	*CPIJ019976*	*3.2094: 4450–9107*
CqUNK_23	midasin	CPIJ010145	3.251: 518823–535901
	centaurin-alpha 2	CPIJ019112	3.1516: 3934–9235
	sterol desaturase, putative	CPIJ009637	3.227: 524825–527033
*CqUNK_28*	*zinc finger protein 40*	*CPIJ018875*	*3.1321: 31848–32423*
	*allatostatin receptor*	*CPIJ016163*	*3.734: 81478–87202*
	*aldehyde oxidase 2*	*CPIJ016888*	*3.821: 149733–154740*
	*bombesin receptor subtype-3*	*CPIJ017637*	*3.980: 85049–99690*
	*defective proboscis extension response, putative*	*CPIJ017115*	*3.897: 12891–19932*
	*40 S ribosomal protein S14*	*CPIJ010397*	*3.291: 138109–144395*
CqUNK_32	choline O-acetyltransferase	CPIJ001609	3.19: 128455–132041
*CqUNK_33*	*phosphatidylinositol-4-phosphate 5-kinase type i*	*CPIJ006826*	*3.145: 280313–292406*
CqUNK_42	Transcription factor Ken 2	CPIJ012629	3.427: 256203–270029
	laminin gamma-3 chain	CPIJ005194	3.96: 903006–924273
	trypsin	CPIJ004641	3.70: 338878–341919
	f-actin capping protein alpha	CPIJ011271	3.322: 247301–257388
	malate dehydrogenase	CPIJ008123	3.169: 118442–121449
*CqUNK_43*	*elongation factor tu*	*CPIJ002277*	*3.30: 1195429–1198055*
	*zinc finger protein*	*CPIJ002883*	*3.37: 1030192–1037437*
	*kakapo*	*CPIJ003239*	*3.41: 533346–586465*
CqUNK_45	monocarboxylate transporter	CPIJ008119	3.184: 607328–610272
	pol-like protein	CPIJ018514	3.1248: 73743–87450
	elongation factor 1 alpha	CPIJ009557	3.231: 370372–372795
	olfactory receptor, putative	CPIJ013754	3.526: 317670–324857

The results summarized in table 3 have been obtained using the elements listed in table 1 as query for BLAST analyses; 84% (313 out of 371) of the insertions detected lay in intergenic regions (i.e. outside the 5 Kbp window upstream/downstream the genes). The remaining 16% (58 insertions) lay in genomic loci where also genes reside (i.e. within 5 Kb upstream/downstream of validated mosquito genes). It is possible that such insertions could contribute to define the spatial and temporal pattern of expression of strictly linked genes.

Among the insertions in proximity of annotated genes, nineteen insertions (5% of the insertions detected) hit genes, and, among them, six insertions (less than 2%) are localized in introns. Standing to the exon-intron organization reported in Vectorbase, the remaining insertions contribute to entire exons or part of them or are localized at exon-intron boundaries. These data suggest that at least a fraction of the LTR retrotransposon insertions that we have considered, could contribute to define the protein-coding regions of genes.

The results obtained using the elements listed in table 2 as query for BLAST analyses indicate that such non-autonomous elements can also be found in genes. Similarly they seem to contribute at the same strength in the building of protein-coding regions of genes in the genome of *C. quinquefasciatus* (table 4). However, after extensive searches against the ESTs databases, we have not been able to find evidences supporting that the retrotransposons analyzed are recruited as exons in the mature transcripts of the genes in which they are inserted. Furthermore, the comparison (not shown) of the genes reported in tables 3 and 4 with the respective orthologs in *Aedes aegypti* suggests that, such insertions are probably recent, and have occurred specifically in the evolutionary lineage of *C. quinquefasciatus*.

These results could be an underestimation, because we intentionally excluded from the BLAST output insertions into, or in proximity to hypothetical protein coding genes that, with the ongoing annotation of the genome could be classified as *C. quinquefasciatus* genes.

## Discussion

In this paper we present data from the LTR_STRUC scan of the *Culex quinquefasciatus* genome. We have been able to identify, by the use of an alternative *in silico* approach, the presence of 67 novel LTR-retrotransposons in the Culex genome. These results contribute to increase the already large dataset of retrotransposons present in the TEfam database. The first consideration to be done is that, in order to identify the repeats complement of a eukaryotic genome the implementation of different methods is necessary. Until now several criteria for the identification of transposable elements have been successfully applied in sequenced genomes. As for the prediction of protein coding genes, two different approaches can be considered for predicting sequences related to transposable element: intrinsic and extrinsic methods. Intrinsic methods allow the identification of transposable elements through identification of genomic sequences having structural properties typical of mobile genetic elements. In contrast extrinsic methods are based on the identification of transposable elements by sequence similarity. It is evident that the latter methods rely on the use of a known transposable element's sequence as query sequence. This constitutes the main limitation of these methods, which makes difficult the identification of novel elements with low sequence similarity respect to the queries. This problem is overcome by the use of intrinsic methods, which look for structures rather than sequence similarity. LTR_STRUC is a program designed for the identification of LTR-retrotransposons [21]. It has been successfully used to identify LTR retrotransposons in mammalian [42] as well as in insect genomes [17] [43]. It is noteworthy that several LTR-retrotransposon finding tools have been recently developed. LTRharvest [22] is a recently described program with best performances respect to other *de novo* finders, including LTR_STRUC. In fact LTRharvest was able to find nearly all the Culex LTR retrotransposons annotated in TEfam, failing in the identification of a single *Ty1/copia*-like element and a single *gypsy*-like element. Furthermore LTRharvest has identified all the elements identified by LTR_STRUC. By contrast the LTR_STRUC program have identified 63/81 Bel/Pao-like elements, 16/32 Ty1/copia-like elements, 44/57 gypsy-like elements. The simplest explanation for the identification of the additional elements in this paper rely into possible differences in the algorithm of different programs or simply because these retrotransposons have been overlooked during former analyses. This underlines the importance of the use of multiple methods, if complex eukaryotic genomic sequences are to be analyzed.

The results obtained integrate the considerably large amount of data existing for mosquitoes' genomes. Indeed, our analyses have uncovered the existence of an additional fraction of the *C. quinquefasciatus* genome related to LTR retrotransposons. This fraction accounts for the 0,8% of the genome occupied by only 29 out of the 67 LTR retrotransposon families detected in this study. In fact, if the non-autonomous elements were also taken in account then this value would have been considerably greater (about 8%).

Our results suggest that a number of LTR retrotransposons insertions could contribute to the built the exon-intron structure of genes in *Culex quinquefasciatus*. Standing to the predicted exon-intron structures of genes in Culex some of the insertions detected could potentially give a contribution in term of exons or parts of them, to the mature form of mRNA expressed from endogenous genes, underlining the importance of retrotransposons and, in general, of mobile elements in shaping the eukaryotic genomes. This aspect could be particularly important for organisms of social relevance, like *C. quinquefasciatus*, because polymorphic TE insertion sites can be at the basis of the resistance emergence that characterize some populations [44]. However we were not able to find ESTs in support of this hypothesis, as well as no homologous genes in related species, such as *Aedes aegypti*, contain retrotransposon related sequences.

Among the novel element identified the vast majority can be classified using conventional criteria, such as combination of phylogenetic clustering and structural features. Unfortunately, these criteria are not sufficient to classify elements lacking coding sequences. This is the case for 38 LTR retrotransposon sequences identified in this study that contain tandemly repeated sequences between LTRs.

Non-autonomous elements lacking ORFs have been well documented especially in plant genomes [45]. Typically, these elements lack all coding sequences but have retained the LTRs, the primer-binding site and the polypurinic tract. These are the minimal features required for replication, because the LTRs contain the promoter needed to produce a template RNA, and the primer-binding site and the polypurine tract are needed to prime the reverse transcription steps. They are extremely heterogeneous in size varying from few hundreds base pairs (TRIM retrotransposons [46] to few Kilobase pairs (LARDS retrotransposons [47]. In mammalian genomes MaLRs retrotransposons (Mammalian Apparent LTR Retrotransposons) [48] have been also described with similar features.

Very interestingly, Arensburger et al. [13] have detected a single element resembling in structure a LARDS retrotransposon in the genome of *C. quinquefasciatus*.

At least two types of observations can be made, looking at the non-autonomous elements described in this paper. First, they apparently lack any functional master copy from which they could have originated. This can be due to the fact that the genome assembly is still in progress or there are genomic regions (such as heterochromatin) that suffer of local low coverage sequencing. The second observation concerns the nature of the repeated sequences, which are not family-specific (i.e. copies belonging to the same family do not share necessarily the same repeat and/or copies of different families could share the same repeat).

It has been suggested that a potential function for the tandem repeats embedded in the LTR retrotransposons could be to facilitate recombination and acquisition of new coding information through gene transduction [49]. A suggestive hypothesis that can be proposed, is that once a LTR retrotransposon acquire, in some way, a repeated sequence it tend to become transpositionally inactive by mean of internal deletions of its coding sequences in *C. quinquefasciatus*. Alternatively, it can be hypothesized that these elements are still capable of transposition if they could use the transpositional machinery of related retroelements *in trans*. In the latter case, the repeated sequences could be disseminated in the genome by passive retrotransposition.

In conclusion, we want to point out that other works have demonstrated the presence of potent regulatory sequences in the repeats carried by retrotransposons, simply by the analysis of their sequence complexity [38] [50]. Similarly, the presence of complex repeats into these non-autonomous elements could be used as starting point to identify similar regulatory elements in *Culex quinquefasciatus*.

In addition, our analysis demonstrates that the genome of *C. quinquefasciatus* contains LTR-retroelements with peculiar features. This was also evident from previous works, which have demonstrated the presence of the *Twin* elements in this genome [13] and have allowed the identification of *Osvaldo*-like elements with a non-canonical structure of the LTRs [17].

In this paper we have also reported the identification of *cqgypsy_1*, an *Osvaldo*-like element with an atypical PBS with a tRNA-dimer structure. The tRNA-dimer is somehow reminiscent of the structure of *Twin* elements described by Feschotte and co-authors. *Twin* has been described as a novel type of SINE element consisting of two tRNA related regions separated by a 39 bp spacer. *Twin* retroelements were found to be abundant in transposon rich genomic regions of *C. quinquefasciatus* [13].

The tandemly repeated tRNAs copies in *cqgypsy_1* display a direct duplication of 26 nucleotides belonging to the 5′LTR.

Indeed, the 26 bp duplication is reminiscent of the target site duplication occurring upon integrase-mediated insertion, suggesting that the tRNA-dimer has been integrated by a transpositional mechanism.

As can be observed from figure 3A both tRNA like sequence halves have the terminal CCA sequences. This structural feature would suggest that the mature form of endogenous tRNA molecules have been incorporated into the retrotransposon backbone after a reverse transcription process. As far as we know, dimerization or aggregation of tRNAs *in vitro* is a known phenomenon, but it typically occurs under non-physiological conditions [51] [52]. On the other hand differences can be highlighted between *Twin* elements and the head to head tRNA repeat found in *cqgypsy_1*. The target site duplication, where it was found, of Twin is an AT rich sequence. A poly-A tract derived from the retroposition event is located downstream *Twin* elements. These features are absent in the *Twin*-like structure that we have detected, suggesting a different origin of the insertion detected in *cqgypsy_1* element.

In conclusion, findings from this and previous reports make *C. quinquefasciatus* a potential niche-genome in which the evolution of transposable elements occurs and generates strong genomic diversity.

The importance of studying the mosquito's *mobilome* also resides in the possibility to use such DNA sequences as molecular biomarkers [53], or for the control of insecticide resistance populations in order to contrast the spread of virus borne diseases [54]. In this view our results could be helpful for future studies concerning such topics.

## Supporting Information

File S1 **DNA sequences of the 67 LTR-retrotransposons identified in this paper.** Each sequence contains 50 bases upstream and downstream allowing unique identification of a reference copy in the genome of *C. quinquefasciatus*.
(TXT)

File S2 **Multiple alignment file used to obtain the phylogenetic tree in** **figure 1** **.**
(TXT)
